# Point-of-care ionised calcium testing: lab validation and clinical feasibility study

**DOI:** 10.1530/EC-25-0337

**Published:** 2025-09-12

**Authors:** Virginia Rozalen Garcia, Tarek Ezzat Abdel-Aziz, Mechteld C de Jong, Francis Lam, Christina Soromani, John Honour, Tom R Kurzawinski

**Affiliations:** ^1^ Centre for Endocrine Surgery, University College London Hospital, London, UK; ^2^ Endocrine Surgery Unit, St. James’s University Hospital, Leeds, UK; ^3^ Biochemistry Department, University College Hospital, London, UK; ^4^ Institute for Women’s Health, University College London, London, UK

**Keywords:** calcium analysis, hypocalcemia, hypercalcemia, hypoparathyroidism, thyroid surgery, parathyroid surgery, point-of-care system, clinical trial

## Abstract

**Background:**

Patients undergoing thyroid and parathyroid surgery require frequent assessment of blood calcium levels to guide their management, and currently such measurements are performed mostly in hospital environments on main laboratory platforms. The aim of this study was to explore the potential use of a non-medical LAQUA device designed to measure ionised calcium in environmental samples as a pocket-size point-of-care device able to measure blood calcium concentration in patients after thyroid and parathyroid surgery.

**Methods:**

The protocol of the study consisted of three distinctive phases: surveying the technological landscape and identifying currently available devices and technologies able to measure calcium in a small volume of whole blood easily, quickly, and accurately (Phase 1); testing the potential candidate device in a laboratory (Phase 2); and performing a prospective, single-arm study (IRAS ID 236079, Protocol number 18/0058, REC ref 19/LO/1740), during which simultaneous calcium measurements were performed on venous and capillary blood on LAQUA and ‘gold standard’ platforms Roche Cobas-Calcium-Gen.2 and Blood Gas Analyser ABL90 (Phase 3).

**Results:**

In Phase 1, LAQUA (HORIBA Inc. Japan) was identified as the most promising potential POC device in terms of size, simplicity of use, and cost effectiveness. In Phase 2, LAQUA showed good accuracy (Δmean = 0.09; *P* = 0.33) and precision (CV 3.41%) in measuring ionised calcium in standardised solutions. In Phase 3, 30 patients were recruited and had 67 sets of measurements. ‘Gold standard’ venous adjusted calcium (Roche) and ionised calcium (BGA) were equivalent, *R* = 0.95, (*P* < 0.001). Strong positive correlation *R* = 0.75 (*P* = <0.001) was observed between venous ionised calcium measured on BGA and LAQUA. Positive but weaker correlation was found between venous (BGA) and capillary (LAQUA) ionised calcium *R* = 0.68, (*P* = <0.001), and between venous and capillary ionised calcium (LAQUA), *R* = 0.56 (*P* = <0.001).

**Conclusions:**

LAQUA is a promising device, which can measure ionised calcium accurately in small venous but not yet capillary blood samples. Further device development is needed before it can be recommended as a potential POC device to measure calcium in blood.

## Introduction

Hypoparathyroidism is a metabolic disorder caused by lack of parathyroid hormone, which leads to hypocalcaemia and hyperphosphataemia. Prevalence of the disease ranges from 6.4 to 37 per 100,000 (1). The estimated prevalence in the European Union is 3.2/10,000 with a growth rate of approximately 0.04 cases/year (2). The majority of cases are post-surgical (3), specifically patients undergoing total thyroidectomy developing temporary (18–27%) or permanent (1–6%) hypoparathyroidism, depending on the series (4, 5, 6). Although categorised as a rare condition, its clinical burden is significant, with 256,000 people with this condition living in the European Union alone (7). There are now several patient advisory and support groups established to help patients cope with symptoms and lifelong implications of having permanent hypoparathyroidism, such as Parathyroid UK (https://parathyroiduk.org/).

Patients with hypoparathyroidism experience a wide range of acute and chronic symptoms, such as muscle cramps, tetany, tachycardia, altered mental status, and soft tissue calcifications. These named symptoms are responsible for decreased quality of life for patients, as well as placing a huge burden on parents and close family members of these patients. This is well recognised in the published literature (3, 8, 9, 10, 11). Hypoparathyroidism can be successfully treated with activated vitamin D analogues and calcium supplements as the primary therapy (12), or replacement therapy with PTH or PTH analogues in selected cases (13, 14, 15, 16).

However, frequent measurement of serum calcium concentration is pivotal in adjusting doses of medications, as both under- and over-treatment can lead to serious side effects and complications. Current guidelines recommend routine biochemical monitoring of albumin-adjusted serum calcium every 3–12 months (7, 17, 18). It is widely acknowledged that as long as calcium is measured in a large sample of venous blood at a main laboratory using photometric methods, this is a practical but unsatisfactory compromise (19).

In response to this urgent and unmet clinical need, our group has embarked on the project of surveying the technological landscape and identifying currently available devices and technologies able to measure calcium in a small volume of whole blood easily, quickly, and accurately (Phase 1), testing the potential candidate device in a laboratory (Phase 2), and performing a prospective, single-arm study of a novel, pocket-size point-of-care (POC) device able to measure blood calcium concentration in patients after thyroid and parathyroid surgery (Phase 3).

## Methods

Phase 1 of the study consisted of a systematic survey of the technological and industrial landscape with the aim of identifying technologies and instruments, which can currently be employed to measure calcium in blood samples of patients with hypoparathyroidism. The survey was performed using Internet searches and a series of interviews with representatives of companies offering such products. Searches were not restricted to equipment licensed for medical use but also included any devices capable of measuring calcium but designed for scientific, domestic, industrial, or environmental use. Potential candidates were ranked according to predefined criteria, taking into account whether they were affordable, sensitive, specific, user friendly, rapid and robust, equipment simple, and could be delivered to end users (20).

Phase 2 of the study was the laboratory testing of the accuracy of a selected device by repeatedly performing measurements of ionised calcium on four identical prototypes using calcium solution concentrations of 0.5, 1, 1.5, and 2 mmol/L, which were pre-prepared in our laboratory. Calibration of the devices was performed as recommended by the manufacturer (https://www.horiba.com/int/veterinary/products/detail/action/show/Product/laquatwin-ca-11c-1-3394/).

Precision was assessed by comparing multiple measurements of the same solution tested with the same prototype/device. Coefficient of variation was calculated (CV = SD/mean).

Accuracy of the prototypes was assessed by comparing the readings each of them presented with the previously known calcium concentration of the solutions. Statistical test: paired Student *t*-test.

Clinical performance was assessed by measuring ionised calcium in venous blood samples of six healthy volunteers using the selected device and the ‘gold standard’ Abbott iSTAT instrument. Performances of both devices were compared using paired Student *t*-test.

Phase 3 of the study was a prospective, clinical, single-arm clinical trial aiming to assess the clinical validity of the selected device to measure ionised calcium in the blood of patients undergoing thyroid or parathyroid surgery during their admission. Ethical approval for the trial was sought and obtained (IRAS project ID 236079, Protocol number 18/0058, REC ref 19/LO/1740), and the trial was registered on a publicly accessible database (Z6364106/2019/07/131; https://www.hra.nhs.uk/).

Patients underwent daily measurements of calcium in venous and capillary blood, both performed sequentially at the same setting. A total of 7–10 mL of peripheral venous blood was collected following venepuncture standard operating procedures. A tourniquet was applied briefly, and butterfly technique and vacutainer/blood gas syringe were used to avoid air exposure. A capillary blood sample was taken immediately after venepuncture using contact-activated lancets (BD Microtainer 2 × 1.5 mm) on patients’ fingertips, and the blood was collected in capillary tubes.

- Venous adjusted calcium was measured on Roche Cobas-Calcium-Gen.2 in the main laboratory using a photometric method, NM-BAPTA at alkaline pH (gold standard I);
- Venous ionised calcium was measured within minutes on Blood Gas Analyser-ABL90 located in the hospital (POCT gold standard II);
- Venous and capillary ionised calcium was measured on LAQUA on the ward using approximately 0.3 μL of venous blood and 1–5 mcl of capillary blood (collected in capillary tubes).

To allow comparison of adjusted and ionised calcium results, equivalence between these measurements was calculated after converting ionised calcium values to adjusted Ca using the normal values ratio (adjCa 2.2–2.6 mmol/L; iCa 1.12–1.32 mmol/L; averaged ratio = 1.97).

Linear correlation between adjusted and ionised calcium in venous and capillary blood was calculated using Pearson correlation coefficient *R*, and agreement between these measurements was assessed by Bland-Altman plots.

Average difference between measurements was calculated as a percentage by comparing the average difference between measurements with the average of ‘gold standard’ calcium measurements.

All statistical analyses were performed using R version 3.5. A *P*-value of <0.05 was considered statistically significant.

## Results

### Phase 1

After considering several devices and various technologies, LAQUA Twin (Horiba Inc. Japan), a simple handheld device designed to measure ionised calcium in solid, viscous, powder, and liquid samples for environmental purposes, was selected for further testing (21).

**Figure 1:**
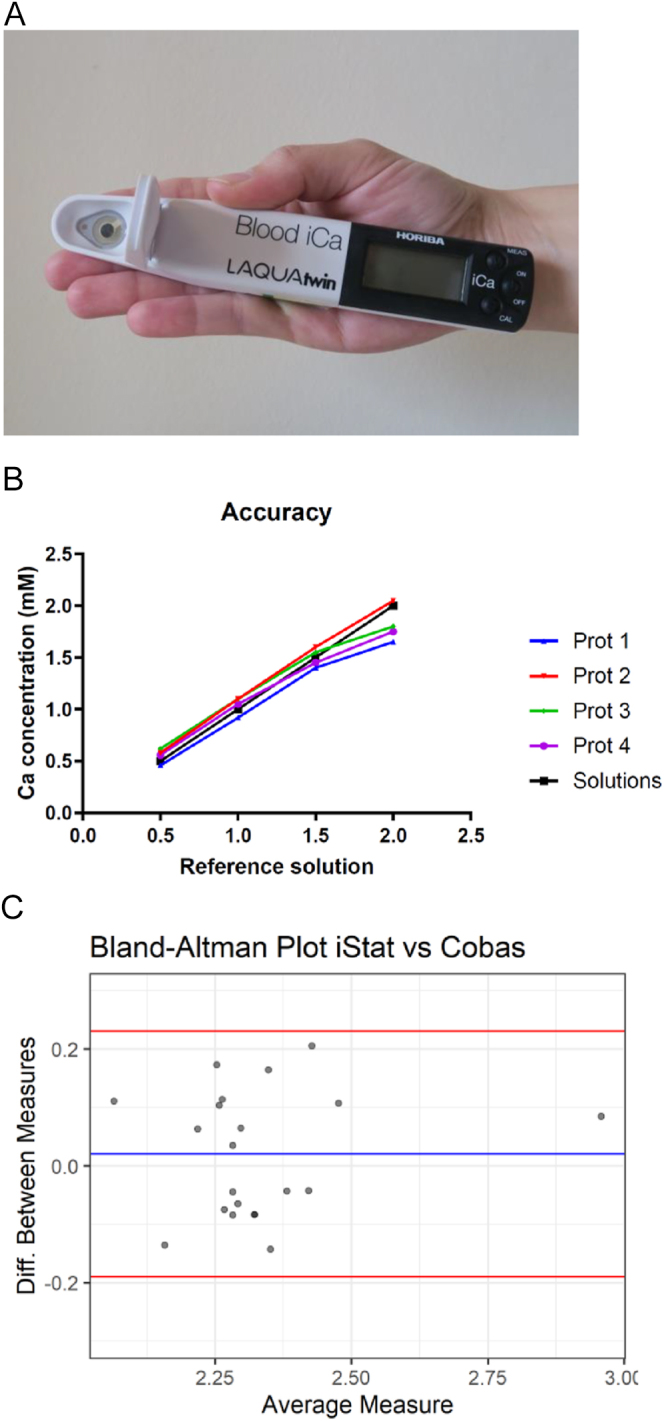
(A) Picture showing LAQUA (Horiba Inc. Japan) prototype, held in hand for scale. (B) Plot showing mean of three measurements in solutions with known calcium concentration using four different LAQUA prototypes (theoretical red line showing 100% concordance). Difference of mean vs reference value = 0.09 (*P* = 0.33). (C) Bland-Altman plot showing difference between measurements by LAQUA prototype and Abbott iSTAT in mmol/L. Blue line shows mean difference, red lines show 95% confidence interval. Δ = 0.05 mmol/L, 95% CI: −0.03–0.13.

LAQUA is a pocket-size (16.5 × 2.8 cm), portable, lightweight device able to measure ionised calcium using an ion-selective electrode and is based on flat sensor technology (Fig. 1A). This device is easy to calibrate and to use, and repeated measurements (up to 1,500) can be performed very fast (5–20 s each) using just a few drops of a sample (0.1–0.3 mL).

### Phase 2

Repeated measurements of ionised calcium on four identical LAQUA devices using pre-prepared calcium solutions (concentrations of 0.5, 1, 1.5, and 2 mmol/L) showed good accuracy (difference of mean vs reference value = 0.09; *P* = 0.33) (Fig. 1B).

Comparison of multiple measurements of the same solution on the same device showed good precision (CV 3.41%), which was similar to the ‘gold standard’ laboratory platform Cobas Calcium Gen 2 (CV 2.5%).

Measurements of ionised calcium in the venous blood from six healthy volunteers on the LAQUA and POCT ‘gold standard’ Abbott iSTAT (mean 1.17; SD ± 0.09 vs 1.21; SD ± 0.04) were not equivalent (*P* < 0.03). However, the difference between the measurements was only 4.5%, which is well within the 10% margin recommended by ISO 15197:2003 when results from a similar point-of-care device measuring glucose are compared to those results obtained on main laboratory platforms (Fig. 1C).

### Phase 3

Thirty patients undergoing thyroid or parathyroid surgery were recruited after giving their consent to participate in the study (Table 1). During their stay in the hospital, patients had a total of 67 sets of daily calcium measurements (as described in Methods above).

**Table 1:** Patient’s characteristics.

Patients characteristics		
Age (mean, range)	52.8 years (20–79)	
Sex (*n*, %)	Female	23 (76.6%)
	Male	7 (23.3%)
Type of surgery	Total thyroidectomy	12 (40.1%)
	Completion thyroidectomy	4 (13.3%)
	Parathyroidectomy	14 (46.6%)

**Figure 2:**
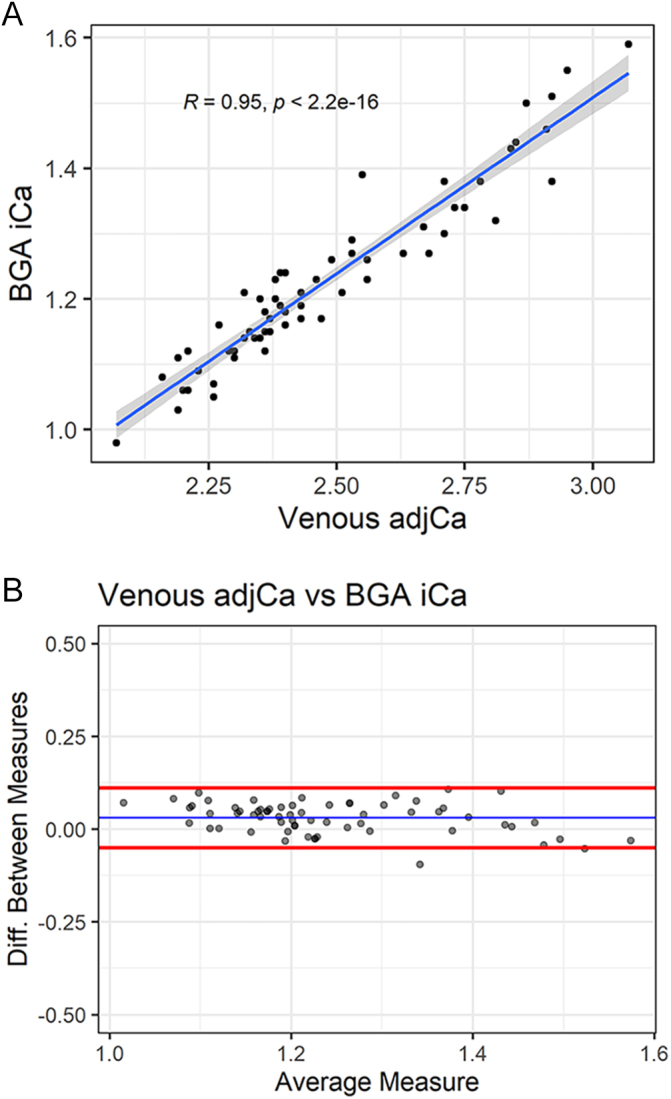
(A) Plot showing venous adjusted calcium measured by Roche Cobas-Calcium-Gen.2, main lab (*x* axis) against venous ionised calcium measured by Blood Gas Analyser-ABL90 (*y* axis) in mmol/L. Blue line shows linear model fitting and confidence interval. *R* = 0.95 (*P* < 0.001). (B) Bland-Altman plot showing difference between measurements of venous adjusted calcium measured by Roche Cobas-Calcium-Gen.2 and ionised calcium by Blood Gas Analyser-ABL90 in mmol/L. Blue line shows mean difference, red lines show 95% confidence interval. Δ = 0.03 mmol/L, 95% CI: −0.05–0.11.

A very strong positive correlation was observed between ‘gold standard I’ venous adjusted calcium (Roche Cobas-Calcium-Gen.2, main lab) and ‘gold standard II’ ionised calcium (Blood Gas Analyser-ABL90) measurements, *R* = 0.95, (*P* < 0.001) (Fig. 2A). Bland-Altman plot showed minimal average difference between measurements (Δ = 0.03 mmol/L, 95% CI: −0.05–0.11) (Fig. 2B). The average difference between gold standard measurements (venous adjCa vs venous iCa BGA) was 2.42%.

**Figure 3:**
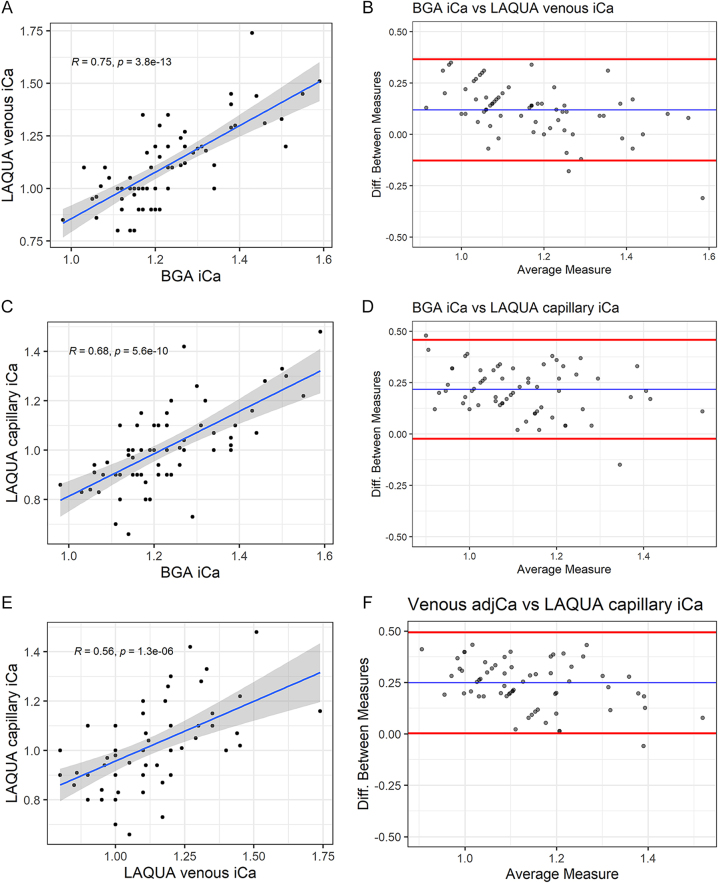
(A) Plot showing venous ionised calcium by Blood Gas Analyser-ABL90 (*x* axis) against venous ionised calcium measured by LAQUA (*y* axis) in mmol/L. Blue line shows linear model fitting and confidence interval. *R* = 0.75 (*P* = <0.001). (B) Bland-Altman plot showing difference between measurements of ionised calcium by Blood Gas Analyser-ABL90 and LAQUA in mmol/L. Blue line shows mean difference, red lines show 95% confidence interval. Δ = 0.14 mmol/L, 95% CI: −0.11–0.41. (C) Plot showing venous ionised calcium by Blood Gas Analyser-ABL90 (*x* axis) against capillary ionised calcium by LAQUA (*y* axis) in mmol/L. Blue line shows linear model fitting and confidence interval. *R* = 0.68 (*P* = <0.001). (D) Bland-Altman plot showing difference between measurements of venous ionised calcium by Gas Analyser-ABL90 and capillary ionised calcium by LAQUA in mmol/L. Blue line shows mean difference, red lines show 95% confidence interval. Δ = 0.22 mmol/L, 95% CI: −0.02–0.46. (E) Plot showing venous ionised calcium by LAQUA (*x* axis) against capillary ionised calcium by LAQUA (*y* axis) in mmol/L. Blue line shows linear model fitting and confidence interval. *R* = 0.56 (*P* = <0.001). (F) Bland-Altman plot showing difference between measurements of venous ionised calcium by LAQUA and capillary ionised calcium by LAQUA in mmol/L. Blue line shows mean difference, red lines show 95% confidence interval. Δ = 0.09 mmol/L, 95% CI: −0.23–0.42.

A strong positive correlation was seen between venous ionised calcium (Blood Gas Analyser-ABL90) and venous ionised calcium (LAQUA) measurements, *R* = 0.75, (*P* < 0.001) (Fig. 3A).

Bland–Altman plot showed just a slightly larger average difference between measurements than in the previous comparison (Δ = 0.14 mmol/L, 95% CI: −0.11–0.41) (Fig. 3B).

The average difference between venous ionised calcium measurements with both platforms was 9.70%.

Similar but slightly weaker correlation was observed between venous ionised calcium (Blood Gas Analyser-ABL90) and capillary ionised calcium (LAQUA), *R* = 0.68, (*P* < 0.001) (Fig. 3C).

Bland–Altman plot showed larger average difference between measurements than seen with venous blood results (Δ = 0.22 mmol/L, 95% CI: −0.02–0.46) (Fig. 3D).

The average difference between venous ionised calcium measurements and capillary ionised calcium was higher as well, at 17.74%.

Finally, we also observed a positive but weak correlation between capillary ionised calcium (LAQUA) and venous ionised calcium (LAQUA), *R* = 0.56, (*P* < 0.001) (Fig. 3E).

Bland–Altman plot showed a small mean difference between measurements. However, there was significant dispersion among measurements (Δ = 0.09 mmol/L, 95% CI: −0.23–0.42) (Fig. 3F).

The average difference between capillary and venous ionised calcium measurements with LAQUA was 8.91%.

## Discussion

Patients and physicians often voiced their concern about the frequency of calcium testing in patients with hypoparathyroidism and called for a change in an old but enduring paradigm based on measuring calcium in a main laboratory using blood samples obtained by venous puncture. Patient advocate groups persistently lobbied for the development of a new device, which would allow patients to perform tests themselves more frequently and conveniently. However, developing diagnostic or therapeutic solutions for patients with rare diseases, which represent small ‘niche markets’, is often not a viable business opportunity for the tech industry. The strategy of repurposing, i.e. adapting existing technologies for a different purpose, is a possible solution to this problem (20).

Our project aimed at identifying and testing an existing commercially available device, which could be repurposed to measure calcium concentration in the patient’s blood quickly and precisely.

LAQUA Twin was identified as the most promising device on the account of its physical properties (size, weight, battery operated), simplicity and speed of the measurement process, and because it employed an ion selective electrode able to measure ionised calcium, an already familiar test in clinical practice. Its ability to measure calcium in very small samples made it attractive as it could facilitate use of capillary blood, an essential requirement for patients performing measurements themselves. Its cost (currently £717.83 GBP) and ability to perform hundreds of tests without expensive disposables suggest it could potentially be very cost effective, with the estimated cost per single measurement at 50 pence. LAQUA has also recently been assessed as a potential cow-side device to measure ionised calcium in bovine blood, against the point of care gold standard (Vet Scan iSTAT) and against the laboratory gold standard (blood-gas analyser-ABL 800 FLEX). The study concluded that LAQUA twin could become a low cost tool for assessing ionised calcium cow-side (22).

Our survey identified many other exciting technologies able to measure calcium; however they were not selected for further testing because of their bulkiness, expense, or difficulty of employing them in POC devices (iSTAT, atomic absorption spectroscopy). Some of them are still being researched and are not currently available commercially (chromophore based spectrophotometric methods, colourimetric nanotechnologies, and wearable electrochemical platforms) but could potentially be refined to the point where they could be employed in clinical scenarios in the future (23, 24, 25).

LAQUA performance in the laboratory showed good accuracy and precision and justified further testing in the blood of healthy volunteers, which confirmed that this device fulfils requirements for POC devices and could be considered for use in clinical scenarios.

The usage of LAQUA has been extensively tested in the final part of our project, which consisted of a trial of LAQUA in a clinical setting. We have chosen to measure calcium in patients undergoing thyroid and parathyroid surgery as the former were expected to have normal calcium before but sometimes low calcium after surgery, and the latter had high calcium before and normal after surgery. Choosing such a population gave us an opportunity to measure calcium within clinically relevant ranges of hypo, normo, and hypercalcaemia.

First, we compared adjusted calcium measured by the laboratory, which is our standard clinical practice, to ionised calcium measured by Blood Gas Analyser-ABL 90, also an approved method of calcium measurement in our hospital. This comparison showed a strong correlation between calcium concentrations using two methods, and by performing this evaluation, we confirmed their equivalence. This observation allowed us to consider BGA ionised calcium as the ‘gold standard’ for comparison with LAQUA measurements, which also assess ionised calcium. It also confirmed that measuring ionised calcium after surgery could be performed in preference to adjusted calcium in the main laboratory, as the results are reliable and instant and clinical decisions need not be delayed. However, this approach is not practical in the hospital setting, as it requires access to BGAs, which are not routinely available on the wards, incurs high costs, and necessitates specialised training (26).

Second, ionised calcium measured by LAQUA and BGA in the same venous sample showed strong positive correlation, but calcium reading values were lower on LAQUA. Similar results were observed in veterinary experiments with bovine blood (22). This indicated some degree of equivalence between measurements, but the difference between measurements (0.14 mmol/L) was larger than when comparing adjusted calcium and ionised calcium measured by BGA (0.03 mmol/L), although within acceptable limits recommended by guidelines for POC devices. This discrepancy is almost certainly the result of the fact that LAQUA is an open system, which affects the stability of the blood. Partial pressure in an open system is lower than in a blood vessel, and this results in CO_2_ being released into the atmosphere once the blood is exposed to air. This results in a change in the pH of blood (alkalinisation) and a decrease in ionised calcium concentration. In addition to air exposure, temperature can also affect ionised calcium concentration, as blood temperature is 37°C and measurements are performed at room temperature. The presence of anticoagulant can also affect calcium measurements.

Third, comparison between BGA venous and LAQUA ionised capillary calcium showed again positive but weaker correlation with a bigger difference in measurements (0.22 mmol/L). This discrepancy between measurements of 0.22 mmol is larger than 10–15% recommended by guidelines for POC devices and will need to be addressed in further studies. We think that this discrepancy is again the result of prolonged exposure of blood to the atmosphere, which is the sum of the time for results to stabilise when in contact with the electrode and the time needed to collect capillary blood, which is longer when capillary blood is collected.

In conclusion, our results confirm that ionised calcium can be used instead of adjusted calcium to assess calcium concentration in patients undergoing thyroid and parathyroid surgery. LAQUA is a precise and accurate novel environmental device, which can be repurposed to measure ionised calcium in small venous blood samples. This device can be used in patients undergoing thyroid or parathyroid surgery, as results can be obtained within seconds at the hospital bedside and facilitate prompt clinical decision making. However, if this technology is to be taken forward to be repurposed for measuring ionised calcium in capillary blood samples to be done at home by patients themselves, future efforts must focus on timing of sample acquisition, its exposure to air, and the temperature at which measurements are performed. This might require changes to the sampling protocol, including use of special porous material to gather capillary blood and/or further development of hardware.

Future studies aiming at developing instruments allowing patients to perform calcium measurements themselves must also address the issue of reliability and accuracy of measurements in the hands of the patients. Such studies should focus on further simplification of the calibration process, ease of use, and maintenance of the device.

## Declaration of interest

The authors declare that there is no conflict of interest that could be perceived as prejudicing the impartiality of the work reported.

## Funding

This study was supported by an Investigator Initiated Research grant from Takeda Pharmaceuticals Internationalhttps://doi.org/10.13039/100016469 AG, a member of the Takeda group of companies (IISR-2017-104126), and a non-restricted grant from Parathyroid UK.

## Ethical approval

Ethical approval for the trial was sought and obtained (IRAS project ID 236079, Protocol number 18/0058, REC ref 19/LO/1740), and the trial was registered on a publicly accessible database (Z6364106/2019/07/131; https://www.hra.nhs.uk/).

## Data availability

Anonymised original data can be made available upon reasonable request.

## References

[1] Bjornsdottir S, Ing S, Mitchell DM, et al. Epidemiology and financial burden of adult chronic hypoparathyroidism. J Bone Miner Res 2022 37 2602–2614. (10.1002/jbmr.4675) 36054571 PMC10087725

[2] Karpf D, Catsburg C & Smith A. Prevalence of hypoparathyroidism in the EU: a systematic review and meta-analysis. Endocr Abstr 2020 70 AEP140. (10.1530/endoabs.70.aep140)

[3] Khan AA, Ali DS, Bilezikian JP, et al. Best practice recommendations for the diagnosis and management of hypoparathyroidism. Metab Clin Exp 2025 171 156335. (10.1016/j.metabol.2025.156335) 40581321

[4] Edafe O, Antakia R, Laskar N, et al. Systematic review and meta-analysis of predictors of post-thyroidectomy hypocalcaemia. Br J Surg 2014 101 307–320. (10.1002/bjs.9384) 24402815

[5] Chadwick D, Kinsman R & Walton P. Fifth National Audit Report of the British Association of Endocrine and Thyroid Surgeons. Henley-on-Thames, UK: Dendrite Clinical Systems Ltd, 2017.

[6] Aspinall S, Mihai R, Kinsman R. BAETS Sixth National Audit Report 2021. Reading, UK: Dendrite Clinical Systems Ltd, 2021. (https://baets.org.uk/reports/)

[7] Bollerslev J, Rejnmark L, Marcocci C, et al. European Society of Endocrinology clinical guideline: treatment of chronic hypoparathyroidism in adults. Eur J Endocrinol 2015 173 G1–G20. (10.1530/eje-15-0628) 26160136

[8] Hypopara UK & Shire. Living with chronic hypoparathyroidism. Meltham, UK: Hypopara UK, 2017. (https://parathyroiduk.org/wp-content/uploads/2018/05/Living-with-chronic-hypoparathyroidism-final-report.pdf)

[9] Hadker N, Egan J, Sanders J, et al. Understanding the burden of illness associated with hypoparathyroidism reported among patients in the paradox study. Endocr Pract 2014 20 671–679. (10.4158/ep13328.or) 24449664

[10] Kiam JS, Sharma V, Glenister L, et al. UK national chronic hypoparathyroidism audit. Clin Endocrinol 2022 97 562–567. (10.1111/cen.14798) PMC9795987 35792134

[11] Vadiveloo T, Donnan PT, Leese CJ, et al. Increased mortality and morbidity in patients with chronic hypoparathyroidism: a population‐based study. Clin Endocrinol 2019 90 285–292. (10.1111/cen.13895) 30375660

[12] Youngwirth L, Benavidez J, Sippel R, et al. Parathyroid hormone deficiency after total thyroidectomy: incidence and time. J Surg Res 2010 163 69–71. (10.1016/j.jss.2010.03.059) 20605611

[13] Tecilazich F, Formenti AM, Frara S, et al. Treatment of hypoparathyroidism. Best Pract Res Clin Endocrinol Metab 2018 32 955–964. (10.1016/j.beem.2018.12.002) 30551988

[14] Clarke BL, Kay Berg J, Fox J, et al. Pharmacokinetics and pharmacodynamics of subcutaneous recombinant parathyroid hormone (1-84) in patients with hypoparathyroidism: an open-label, single-dose, phase i study. Clin Ther 2014 36 722–736. (10.1016/j.clinthera.2014.04.001) 24802860

[15] Cusano NE, Rubin MR & Bilezikian JP. PTH(1-84) replacement therapy for the treatment of hypoparathyroidism. Expert Rev Endocrinol Metab 2015 10 5–13. (10.1586/17446651.2015.971755) 25705243 PMC4334142

[16] Bilezikian J, Khan A, Potts J, et al. Hypoparathyroidism in the adult: epidemiology, diagnosis, pathophysiology, target organ involvement, treatment, and challenges for future research. J Bone Miner Res 2011 26 2317–2337. (10.1002/jbmr.483) 21812031 PMC3405491

[17] Khan AA, Koch CA, Van Uum S, et al. Standards of care for hypoparathyroidism in adults: a Canadian and International Consensus. Eur J Endocrinol 2019 180 P1–P22. (10.1530/eje-18-0609) 30540559 PMC6365672

[18] Brandi ML, Bilezikian JP, Shoback D, et al. Management of hypoparathyroidism: summary statement and guidelines. J Clin Endocrinol Metab 2016 101 2273–2283. (10.1210/jc.2015-3907) 26943719

[19] Caprita R, Caprita A & Cretescu I. Estimation of ionized calcium and corrected total calcium concentration based on serum albumin level. Anim Sci Biotechnologies 2013 46 180–184.

[20] St John A & Price CP. Existing and emerging technologies for point-of-care testing. Clin Biochem Rev 2014 35 155–167. 25336761 PMC4204237

[21] Goulet EDB & Asselin A. Reliability and validity of a low cost, pocket-sized and battery operated sodium analyzer in measuring urinary sodium concentration. Technol Health Care 2015 23 881–891. (10.3233/thc-151028) 26409516

[22] Neves RC, Stokol T, Bach KD, et al. Method comparison and validation of a prototype device for measurement of ionized calcium concentrations cow-side against a point-of-care instrument and a benchtop blood-gas analyzer reference method. J Dairy Sci 2018 101 1334–1343. (10.3168/jds.2017-13779) 29248221

[23] Guo Z, Johnston WA, Stein V, et al. Engineering PQQ-glucose dehydrogenase into an allosteric electrochemical Ca^2+^ sensor. Chem Commun 2016 52 485–488. (10.1039/c5cc07824e) 26528736

[24] Nyein HYY, Gao W, Shahpar Z, et al. A wearable electrochemical platform for noninvasive simultaneous monitoring of Ca^2+^ and pH. ACS Nano 2016 10 7216–7224. (10.1021/acsnano.6b04005) 27380446

[25] Kim S, Park JW, Kim D, et al. Bioinspired colorimetric detection of calcium(II) ions in serum using calsequestrin-functionalized gold nanoparticles. Angew Chem Int Ed 2009 48 4138–4141. (10.1002/anie.200900071) 19425025

[26] Mirzazadeh M, Morovat A, James T, et al. Point-of-care testing of electrolytes and calcium using blood gas analysers: it is time we trusted the results. Emerg Med J 2016 33 181–186. (10.1136/emermed-2015-204669) 26396233

